# How many fathers? Study design implications when inferring multiple paternity in crocodilians

**DOI:** 10.1002/ece3.9379

**Published:** 2022-10-05

**Authors:** Sally R. Isberg

**Affiliations:** ^1^ Centre for Crocodile Research Noonamah Northern Territory Australia

**Keywords:** *Alligator*, *Caiman*, crocodilian, *Crocodylus*, microsatellites, multiple paternity

## Abstract

Up to 10 males were reported to sire clutches of crocodilian eggs but review of the underlying study designs raised questions of potential upward bias of inferred sire numbers. To test this premise, different scenarios were explored using a published dataset of 16 known single‐sire saltwater crocodile pairs and their offspring which were originally confirmed using a 11 loci microsatellite panel in CERVUS. Varying the number of microsatellites, omitting one or both parental genotypes and using different parentage analysis techniques revealed that total allele number, rather than number of loci, determined inferred sire accuracy in two opposing ways. Using the single‐locus minimum method and GERUD, which both require prior knowledge of family groupings (i.e., nests), fewer alleles (and loci) accurately inferred only one father. In contrast, CERVUS and COLONY required all 11 loci (65 alleles) and both parental genotypes to (a) assign correct family groups and (b) infer the correct sire number, except in one family where two sires were equally assigned based on their number of homozygous loci. When less genotype information was provided, CERVUS and COLONY inferred up to six and seven sires, respectively. Given this data is from confirmed single‐sire matings, and yet up to seven sires could be inferred, the significance of inappropriate study design is clearly demonstrated. Consideration should be carefully given to genotype data, particularly those collected specifically for population diversity studies, which are also used to infer multiple paternity because the underlying data collection assumptions are not equivalent between the two outcomes.

## Graphical abstract

Up to ten males have been reported to sire clutches of crocodilian eggs but review of the underlying study designs raised questions of potential upward bias of inferred sire numbers. Using a known dataset from 1:1 male:female matings, an impossible 7 sires were inferred by limiting the genotypic information using a particular statistical package proving the potential upward bias of inferred sire numbers possible. To avoid this bias, authors are encouraged to maximise the genotypic information available, test different scenarios using the multiple analytical methods available and to report on the lowest number of inferred sires remembering these sires are hypothetical until proven by true genotype.

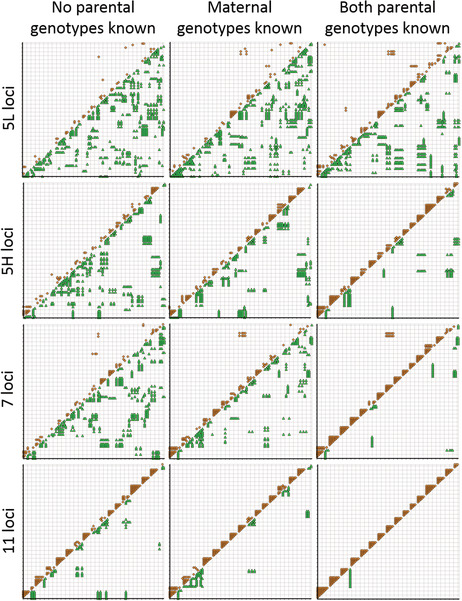

## 1 INTRODUCTION

Multiple paternity in crocodilian species is now well‐established (Isberg, 2020). Twelve of the 25 extant crocodilians have shown the potential for multiple paternity, as summarized in Table 1, with paternity studies on the other species yet to be conducted but likely to yield the same conclusion. Most of these studies had a primary aim of describing mating systems and levels of transferred heterozygosity to the offspring population to understand and enhance the success of conservation programs by ensuring genetic diversity. To derive this information, the majority of these studies collected hatchli DNA samples from hatchlings from different clutches of eggs. In some cases, maternal or likely maternal samples were retrieved as the eggs were collected, and in other cases a pool of adult samples from the same population (candidate parents) might also be available. Therefore, it would be seemingly logical to also use these data to test the hypothesis of multiple paternity. However, the lack of proven utility of the selected microsatellite loci panels as applied to known pedigree structures begs the question of whether the null hypothesis (ie no multiple paternity) has been properly evaluated, or if the number of sires being reported are fabrication of the statistical methodology being employed in combination with the arbitrary utility of microsatellites selected.

**TABLE 1:** Summary of the study designs, microsatellites, parentage assignment methods, and reported outcomes that are used to detect multiple paternity in crocodilians

Species/reference	Sampling information				Microsatellite information				Reported outcomes			
	No. of nests	No. of hatchlings sampled/clutch (%)	No. of candidates sampled		No. loci	Of Range of allele number (total)	Range (average)		Parentage assignment method	Derived no. sires/nest	Rate of multiple paternity	Contribution of primary male
			Dams	Sires			H_obs_	H_exp_				
Alligatoridae												
*Alligator mississippiensis*												
Davis et al. (2001)	22	14–40 (100%)	22	0	5	5–16 (44)	0.49–0.83 (0.68)	0.49–0.86 (0.72)	DeWoody et al. (2000)	1–3	32%	47%^a^
Lance et al. (2009)	114	19.6^b^ (100%)	NR	0	5	9–19 (54)	NR	NR	GERUD 2.0	1–3	51%	NR
Zajdel et al. (2019)	151	14.6^c^ (100%)	120	84	5	9–20 (67)	0.67–0.84 (0.79)	0.67–0.85 (0.80)	COLONY	1–3	43%	NR
*Alligator sinensis*												
Hu and Wu (2010)	10	8–18 (100%)	10	0	5	4–6 (24)	0.33–0.78 (0.64)	0.62–0.80 (0.69)	CERVUS 2.0	>2	33%	NR
*Caiman crocodilus*												
Oliveira et al. (2010)	13	NR (30–100%)	11	21	6	NR	NR	NR	SLMM & GERUD 2.0	2–4	100%	NR
Oliveira et al. (2014)	20	3–16 (<30%)	13	0	6	11–22 (91)	NR	0.81–0.87 (0.85)		1–4	95%	NA^d^
*Caiman latirostris*												
Amavet et al. (2008)	4	10–15	4	0	8	NR	NR	NR	SLMM, CERVUS 3.0 & GERUD 2.0	1+	50%	NA^d^
Amavet et al. (2012)	12	9–16	12	0	4	5–7 (24)	0.11–0.60 (0.44)	0.70–0.77 (0.75)		>2	17%	NA^d^
*Caiman yacare*												
Ojeda et al. (2017)	13	7–12 (30–40%)	0	0	7^i^	2–10 (42)	0.15–0.49 (0.34)	0.22–0.50 (0.33)	GERUD 2.0	1–3	67%	NA^d^
*Melanosuchus niger*												
Santos et al. (2010)	10	13–35 (100%)	0	0	5	NR	NR	NR	NR	1+	90%	NR
Muniz et al. (2011)	6	5–6	0	0	5	2–7 (26)	NR	NR	SLMM & COLONY	1+	33%^e^	NA^d^
Crocodylidae												
*Crocodylus acutus*												
Budd et al. (2015)	NA^f^	NA^d^	0	0	9	NR	NR	NR	KINGROUP	1–2	NR	NR
*Crocodylus intermedius*												
Rossi Lafferriere et al. (2016)	20	3–42 (100%)	0	0	17	2–11 (90)	0.14–0.82 (0.52)	0.14–0.86 (0.54)	COLONY & GERUD 2.0	1–3	50%	57–95%
*Crocodylus moreletii*												
McVay et al. (2008)	10	NR (100%)	0	42^g^	6^j^	3–7 (26)	0.41–0.80 (0.48)	0.41–0.74 (0.69)	GERUD 2.0	1+	50%	4:1
*Crocodylus niloticus*												
Nöthling et al. (2020)	25	4–6	0	0	11	3–20 (75)	0.35–0.71 (0.54)	0.47–0.82 (0.64)	SLMM & COLONY	1–2+	52%–76%	NR
*Crocodylus porosus*												
Lewis et al. (2013)												
Wild	13	29.7^b^	0	0	5	3–10 (32)	0.51–0.83 (0.68)	0.53–0.81 (0.68)	PARENTAGE 1.0 & CERVUS 3.0	1–4	69%	NR
Captive	21	17.3^b^	2	12		5–9 (35)	0.49–0.86 (0.70)	0.58–0.82 (0.73)		1–10	43%^h^	NR
*Crocodylus rhombifer*												
Milián‐García et al. (2016)	5	11–31 (100%)	65	50	9^k^	2–13 (68)	0.07–0.70 (0.55)	0.11–0.78 (0.60)	COLONY	2–6	100%	44%–79%

Abbreviations: NR, not reported; NA, not applicable; H_obs_ and H_exp_, observed and expected heterozygosity, respectively; SLMM, single‐locus minimum method of Myers and Zamudio (2004).

^a^ One clutch only.

^b^ Average number of hatchlings per nest.

^c^ Calculated from years when total clutches were collected.

^d^ Cannot be calculated as not all offspring were genotyped.

^e^ From simple allelic count.

^f^ Hatchlings were caught after emergence from the nest.

^g^ Total candidate parents genotyped.

^h^ Recalculated from Lewis et al. (2013).

^i^ Only 7 loci were used.

^j^ Excludes loci with only one allele.

^k^ Calculated by SRI using Cervus 3.0 (Kalinowski et al., 2007).

Multiple paternity in crocodilians was first reported by Davis et al. (2001) in American alligators (*Alligator mississippiensis*). The study design strategically selected nests based on the presence of a female displaying nest‐guarding behavior (presumed maternity) and being able to obtain a blood sample (to confirm maternity). Of the twenty‐two nests collected, all of the female genotypes were aligned with the resultant offspring from each nest (i.e., true dams). Seven of these nests displayed evidence of multiple paternity with up to three possible sires.

Of the studies conducted since then, up to 10 males were inferred to sire captive saltwater crocodile (*Crocodylus porosus*) nests (Lewis et al., 2013) although 1–3 sires are most commonly reported (Table 1). Further, between 17% (Amavet et al., 2012) and 100% (Milián‐García et al., 2016) of nests were shown to be multiply sired. Some studies have used a small subset of nests from only one nesting season (eg *n* = 4 nests from Amavet et al., 2008) while others have conducted longitudinal studies over multiple years (Lance et al., 2009) including up to 151 nests over six consecutive years (Zajdel et al., 2019). Most of the studies have recovered DNA from all resultant hatchlings from a nest, although in real terms this has sometimes only been three hatchlings (Rossi Lafferriere et al., 2016). In contrast, other studies limited their offspring genotyping to less than 30% of total hatchlings (3–16 offspring/clutch; Oliveira et al., 2014). Of the 17 studies, the majority had candidate maternal genotypes available but there are seven studies that do not have any parental genotypes available. To assess these mating systems, between four and 17 microsatellites were employed with allele numbers ranging between two and 22. Various parentage assignment methods are used, although the most popular are GERUD 2.0 (Jones, 2005), COLONY (Wang, 2016), and the single‐locus minimum method (SLMM) described by Myers and Zamudio (2004).

As such, varying study designs and lack of null hypothesis testing lead to the question of potential upward bias based on the differences in predictive power, on the accuracy of inferring multiple paternity and number of contributing sires particularly in the absence of parental genotypes. To assess these implications, the data from Isberg et al. (2004) were re‐analyzed using different scenarios that emulate a range of potential study designs incumbent with wild population studies. Isberg et al. (2004) showed the utility of 11 microsatellite markers to assign parentage of *C. porosus* in the context of improving the accuracy of genetic improvement programs. Sixteen known‐breeding pairs (one male and one female) that were housed together for numerous years were genotyped. One hundred and seven offspring were retrospectively identified using clutch‐specific scute cuts (Isberg et al., 2005) and genotyped. Using CERVUS 2.0 (Marshall et al., 1998), a pedigree error rate of 5.6% (*n* = 6) was detected due to either wrong scute cutting at time of hatch or mis‐reading the scute cuts during sampling. By excluding these six individuals, the remaining 101 offspring along with the known and confirmed genotypes of both sire and dam with no possibility of cross‐mating represent the ideal dataset to simulate different study designs that could affect the number of crocodilian sires being inferred from wild populations.

Using the known dataset from Isberg et al. (2004), this study hypothesized that by limiting different elements of information provided to the different parentage assignment software used, the number of inferred sires could be appreciably upward biased. This included varying the number of microsatellites evaluated, excluding population allele frequencies and excluding parental genotypes. These same limitations were also applied when assigning sibship (family groups) where the hypothesis was that the less information provided, the more error in assigning familial groupings would result.

There is also a future possibility to test the underprediction of sire number by combining some of these known family groups and replacing genotypes to simulate multiple paternity. However, this was not pursued in this study.

## 2 METHODS AND MATERIALS

### 2.1 Data set

The genotypes from 16 sires and 16 dams, which were housed as long‐term one male: one female breeding pairs from Janamba Crocodile Farm, Middle Point, Northern Territory, Australia were used as described by Isberg et al. (2004). These adult *C. porosus* were originally sourced from the wild as part of the problem crocodile program around the Darwin region (Fukuda et al., 2014). Allele frequencies for the 11 microsatellites were developed by FitzSimmons et al. (2001) and found to be useful for parentage assignment as shown in Table 2. The 101 offspring of confirmed parentage aligning to these 16 breeding pairs were used herein. The average number of offspring per family was 6 (range 4–9).

**TABLE 2:** Summary of microsatellite loci trialed on 32 adult *C. porosus* as described by Isberg et al. (2004)

Loci	No. alleles	Range of allele sizes (base pairs)	H_obs_	H_exp_	Microsatellite panel			
					11 loci	7 loci	5H loci	5L loci
Cj127	16	353–415	0.813	0.861	Y		Y	
Cj131	8	228–242	0.875	0.82	Y		Y	
Cj101	6	345–367	0.625	0.707	Y	Y	Y	
CUD68	6	137–147	0.563	0.58	Y	Y	Y	
Cj16	6	156–187	0.719	0.603	Y	Y	Y	Y
Cj18	5	185–228	0.75	0.769	Y	Y		Y
Cj105	4	365–371	0.563	0.488	Y	Y		Y
Cp10	4	196–204	0.594	0.675	Y	Y		Y
Cj119	4	178–188	0.594	0.66	Y	Y		Y
Cj104	3	206–210	0.813	0.616	Y			
Cj122	3	375–387	0.219	0.201	Y^a^			
Number of identical pairwise genotypes					3	9	13	21
Number of alleles					65	35	42	23

*Note*: H_obs_ and H_exp_ are observed and expected heterozygosity, respectively. The loci used in each microsatellite panel to test the robustness of study design are also indicated. 5H and 5L designed 5 loci with high (H) and low (L) polymorphic information content.

^a^ Indicates this microsatellite was not used in the GERUD 2.0 analysis as the program is limited to 10 loci (Jones, 2005). The number of identical pairwise genotypes are given for each microsatellite panel.

### 2.2 Microsatellite panels

Eleven microsatellites were used in Isberg et al. (2004) to confirm parentage of these offspring. To compare the added value of so many microsatellites, scenarios using a reduced number of microsatellites were compared to the full complement. Seven and five loci were chosen as the reduced microsatellite panels as they represent the average and modal number of microsatellites used in the crocodilian multiple paternity literature to date (Table 1). The seven‐locus panel was constructed by removing the two least and the two most polymorphic loci from the full complement panel (Table 2). Two five microsatellite panels were constructed based on polymorphic information content: the five most polymorphic loci were included in the five high (5H) loci panel, and five least polymorphic were included in the five low (5L) loci panel, as indicated in Table 2.

As reported within Isberg et al. (2004), the genotype frequencies of the adult animals were within expectations of Hardy–Weinberg equilibrium at each locus (*p* > .05), with the exception of Cj104 (*p* = .032). However, this locus was kept in the analysis because minor deviations from Hardy–Weinberg equilibrium at few loci are unlikely to bias likelihood estimates considerably across all loci (Marshall et al., 1998). In addition, because only 32 wild‐caught adults from various locations were sampled, these animals may not represent a sample from the overall *C. porosus* population. As such, no corrections were required.

### 2.3 Parentage assignment software and scenarios for comparison

#### 2.3.1 CERVUS 3.0

Jones et al. (2010) created a decision‐making flowchart to choose the most appropriate parentage analysis software based on available genotypes and sampling schemes. On the basis of the information available from Isberg et al. (2004), that is parentage genotypes are available but mean family size is less than eight, the most appropriate parentage analysis technique is exclusion/allocation or a full probability model. CERVUS 3.0 (Kalinowski et al., 2007) uses an exclusion/allocation technique by calculating a LOD score (logarithm of the likelihood ratio) for each offspring–parent pairing. One advantageous feature of CERVUS 3.0 is the likelihood expressions incorporate a genotype replacement model that accounts for genotypic mismatches in the dataset from mutations or experimental error. An earlier version of CERVUS 2.0 (Marshall et al., 1998) was originally used by Isberg et al. (2004). This program was again used, with the full and reduced microsatellite panels, to simulate two scenarios for parentage assignment: (1) identifying the male parent when the female is known and (2) identifying either parent with no prior knowledge of the other. Once the first parent was identified in the second scenario for each offspring, the most common parent for each family group was set as the known parent, and the analysis used to re‐run the first scenario specifying all other parental genotypes as the potential second parent (i.e., the sexes were not specified). CERVUS 3.0 needs to calculate population allele frequencies for each microsatellite loci before simulations of parentage testing can be conducted.

#### 2.3.2 GERUD

If the Isberg et al. (2004) data had a mean family size greater than 8–10 offspring in addition to the pool of parental genotypes, Jones et al. (2010) recommended parental reconstruction (e.g., GERUD 2.0; Jones, 2005) augmented by either exclusion/allocation (e.g., CERVUS 3.0) or full probability modeling. GERUD 2.0 is the most commonly used parentage analysis software used in the crocodilian literature (Table 1). GERUD 2.0 combines progeny array genotypes using an exhaustive algorithm to reconstruct the minimum number of parents to deduce multiple paternity without the need to specify population allele frequencies (Jones, 2005). Scenarios specifying both known and unknown maternal genotypes were evaluated. GERUD 2.0 is limited to 10 microsatellite markers, so for this study, the least informative loci (Cj122) was not included in the full microsatellite panel analysis as indicated in Table 2. Unlike CERVUS, GERUD 2.0 cannot elucidate genotypic mismatches in the dataset from mutations or experimental error.

#### 2.3.3 COLONY

Jones et al. (2010) recommended the use of sibship reconstruction when family (half‐ and full‐sib) groups cannot be identified *a priori*. As crocodiles lay clutches of eggs (ie family groups), they do not require sibship reconstruction yet COLONY is the second most common parentage analysis software used in crocodilian parentage assignment (Table 1). COLONY uses a maximum‐likelihood pedigree analysis to assign individuals into full‐ or half‐sib arrays (Wang, 2016). Possible parental genotypes can be specified so three scenarios were compared: (1) the maternal genotypes are known, (2) both parental genotypes are known, and (3) no parental genotypes are known. In addition to specifying parental genotypes, the estimated proportion of parents genotyped can be specified so scenarios of 25%, 50%, and 100% genotyped were compared. Finally, comparing the improved accuracy of including population allele frequencies was also evaluated. Allele frequencies estimated from the CERVUS 3.0 analyses were used. In all scenarios, 10 replicate runs of “long” length employing a full‐likelihood method of “high” precision were specified assuming an error rate of zero for allelic drop‐out and 5% for genotyping error. Although these offspring were from known breeding pairs under the multiple paternity scenarios being tested, polygamy was specified for both sires and dams.

#### 2.3.4 Single‐locus minimum method (SLMM)

This technique is the least attractive due to the manual counting of paternal alleles at each locus within a family group, and dividing by two (Myers & Zamudio, 2004). This rudimentary technique does not account for multilocus allele associations or population allele frequencies but was of interest in comparing the results of the more computationally challenging techniques.

### 2.4 Comparing outcomes from parentage assignment software

For each scenario described above, there were three parameters of interest:

1. Number of inferred sires – For each of the 16 family groups, the number of inferred sires were counted, and a mean and maximum number of inferred sires was reported,
2. Family assignment – Irrespective of number of inferred sires, the number of family groups correctly assigned as having one common parent was counted (CERVUS 3.0 and COLONY only), and
3. Pair assignment – Given these data are from 16 known pair matings, the number of correct family groups with one male and one female were also counted (CERVUS 3.0 and COLONY only).

## 3 RESULTS

### 3.1 Identity analysis

The full 11 loci microsatellite panel had a total of 65 alleles compared to the reduced panels of 7, 5H, and 5L which had 35, 42, and 23, respectively (Table 2). Both CERVUS 3.0 and COLONY can inform the user of the number of identical genotypes in a dataset. As the number of microsatellite loci (and alleles) decreased, the number of identical pairwise genotypes increased ranging from three (i.e., 6 individuals) when 11 microsatellites were used to 21 (32 individuals; 24% of total population) when the 5L microsatellite panel was used (Table 2).

### 3.2 Number of inferred sires

**FIGURE 1:**
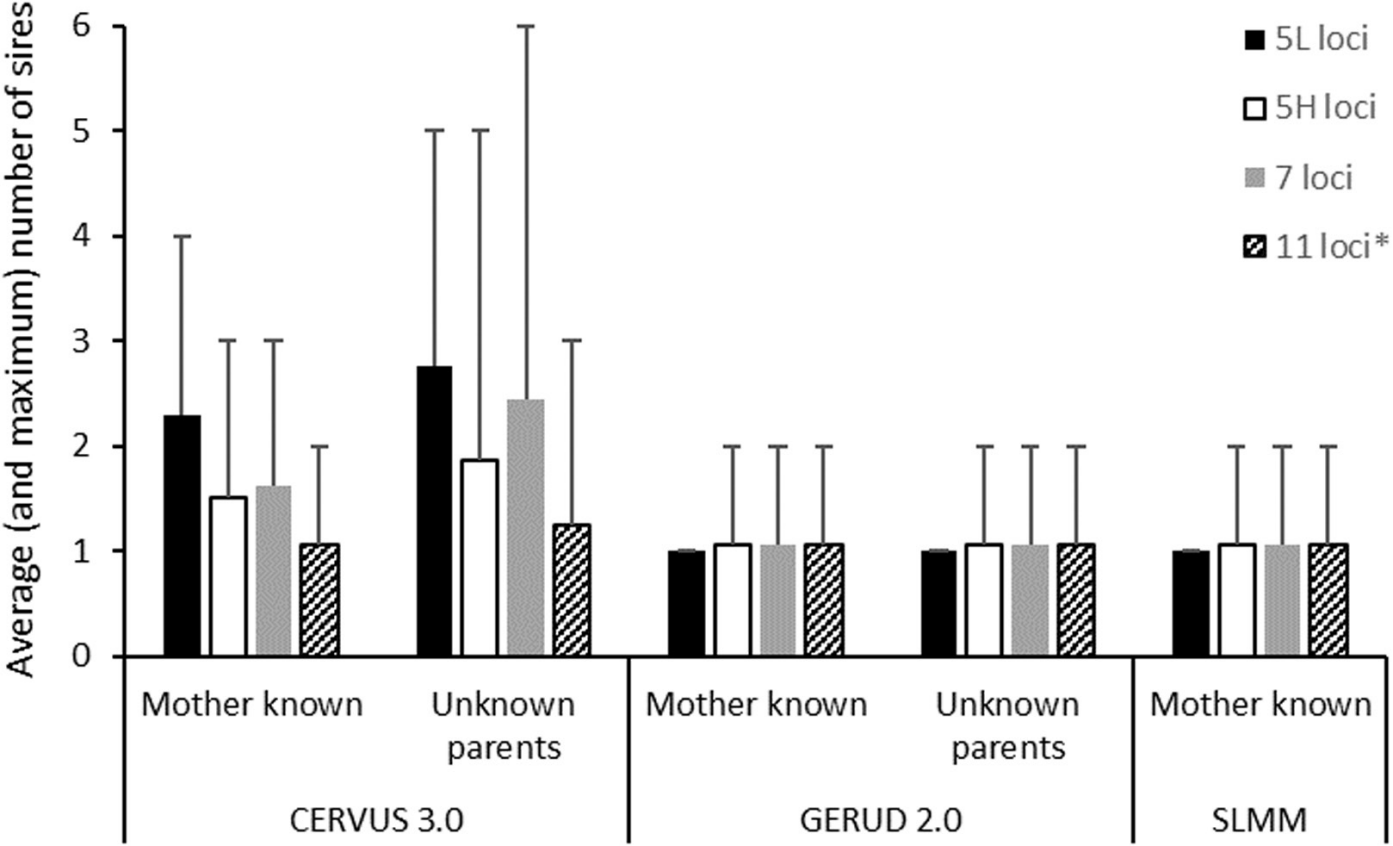
Average (and maximum) number of sires inferred per family using Cervus 3.0 (Kalinowski et al., 2007) and GERUD 2.0 (Jones, 2005) when the maternal genotype was both known and unknown. For the single‐locus minimum method (SLMM; Myers & Zamudio, 2004), only results with known maternal genotypes can be reported as the method requires prior knowledge of the maternal family group. Four microsatellite panels were compared: 5 loci with low polymorphism (5L; solid black), 5 loci with high polymorphism (5H; white), 7 loci (gray) and 11 loci (diagonal lines). *GERUD 2.0 restricts the maximum number of loci to 10.

**FIGURE 2:**
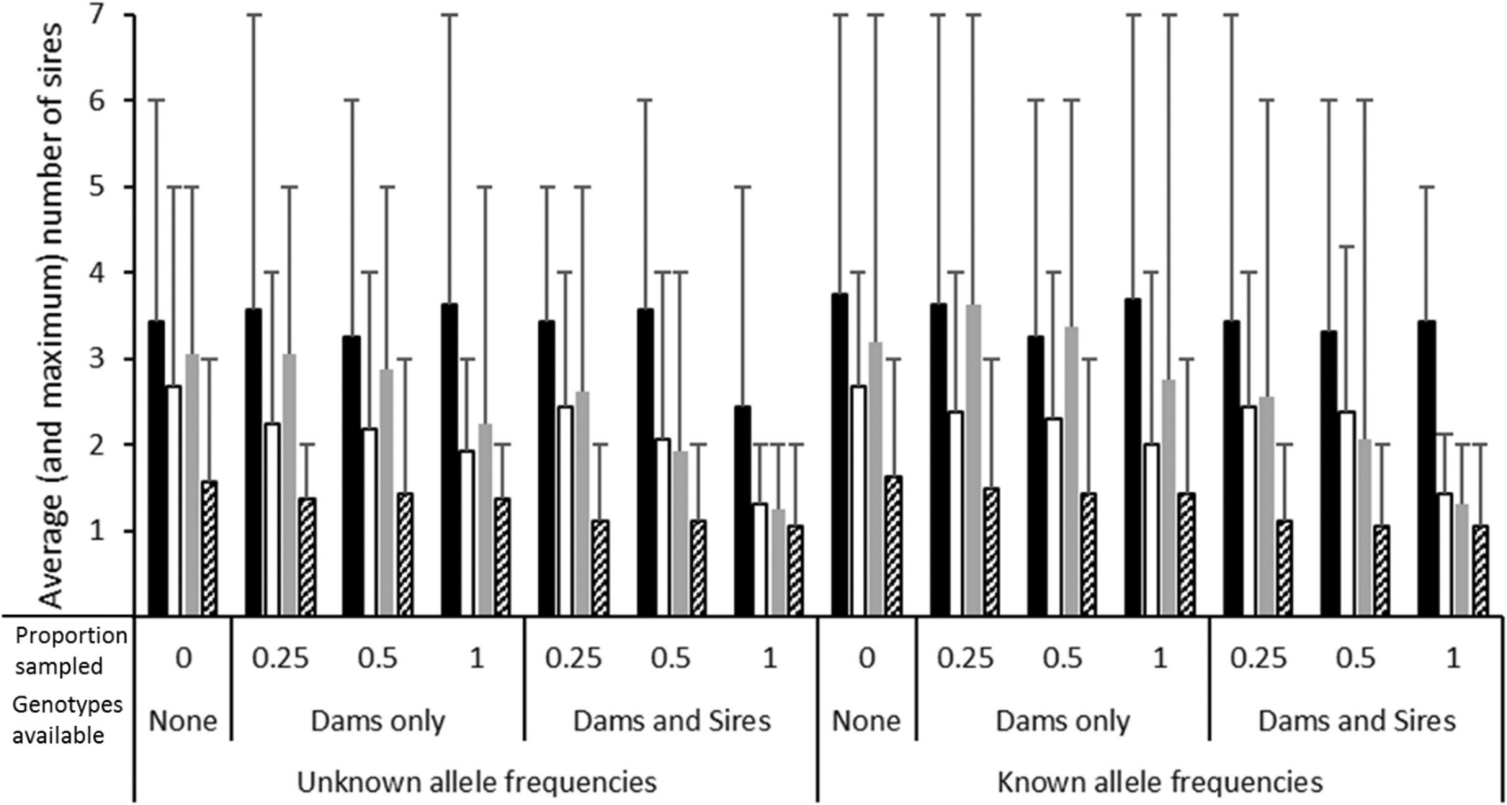
Average (and maximum) number of sires inferred per family using COLONY (Wang, 2016) comparing the inference when allele frequencies were either known or unknown, depending on the type of genotype data available (none, dams only, or both dams and sires) as well as the proportion of the candidate parent population genotyped. Four microsatellite panels were compared: 5 loci with low polymorphism (5L; solid black), 5 loci with high polymorphism (5H; white), 7 loci (gray) and 11 loci (diagonal lines).

The number of sires per family inferred by CERVUS 3.0, GERUD 2.0, and the SLMM are shown in Figure 1 and those inferred by COLONY are shown in Figure 2. Of all the analyses, the correct number of sires (i.e., one per family) was only inferred using the low polymorphic 5 (5L) loci microsatellite panel employing either SLMM or GERUD 2.0 regardless of whether maternal genotypes were known, although fewer possible paternal genotype reconstructions resulted when the maternal genotypes were specified. For the other microsatellite panels, GERUD 2.0 and SLMM had an average of 1.1 sires per family due to one family group (B1) inferring two sires based on a presumed mistyping error of one offspring (offspring ID 101) at locus Cj101 (allele 359). CERVUS 3.0 also inferred 1.1 sires per family using the 11 loci microsatellite panel when the maternal genotype was known (Figure 1) after inferring two sires in a different family (B3) to that of GERUD 2.0 and SLMM. In contrast to GERUD 2.0 and SLMM, using the reduced microsatellite panels in CERVUS 3.0 increased the average and maximum number of inferred sires. When only maternal genotype was known, the 5L panel inferred four sires per family group and the 5H and 7 loci panels inferred three sires. When neither parent was known, CERVUS 3.0 inferred up to three sires per family, with an average of 1.25, when 11 microsatellite loci were used. However, using the reduced microsatellite panels up to six sires/family (average 2.4) were inferred using 7 loci.

Using COLONY, the lowest average number of sires was 1.1 (maximum 2) using the 11‐microsatellite panel when a pool of candidate sire and dam genotypes were available, irrespective of the proportion sampled or whether allele frequencies were specified (Figure 2). When the same 11 microsatellites were used but only candidate dams were provided, up to three sires per family could be inferred (average = 1.4). The 5L loci panel inferred the highest average number of sires (3.4 sires/family) and the 7 loci panel inferred the second highest number (2.6 sires/family). Both of these panels returned up to seven sires per family group.

Although COLONY allows the user to specify allele frequencies or not, inclusion did not change the number of inferred sires in 57% of scenarios but did overinflate sire number in 39% by up to two sires. For example, the number of sires increased from five to seven using the 7 loci panel when the proportion of genotyped dams were specified as both 25% and 100% as well as when the 5L loci panel was used and 25% of both dam and sire genotypes were available (Figure 2). In contrast, providing COLONY with a higher proportion of candidate parent genotypes deflated the number of inferred sires. The largest difference was a decrease from six inferred sires using the 7 loci panel with known allele frequencies but stipulating only 25% of known parental genotypes to two inferred sires by stipulating that 100% of the parental population was genotyped, a difference of four inferred sires.

### 3.3 Proportion of correct family groups assigned

**FIGURE 3:**
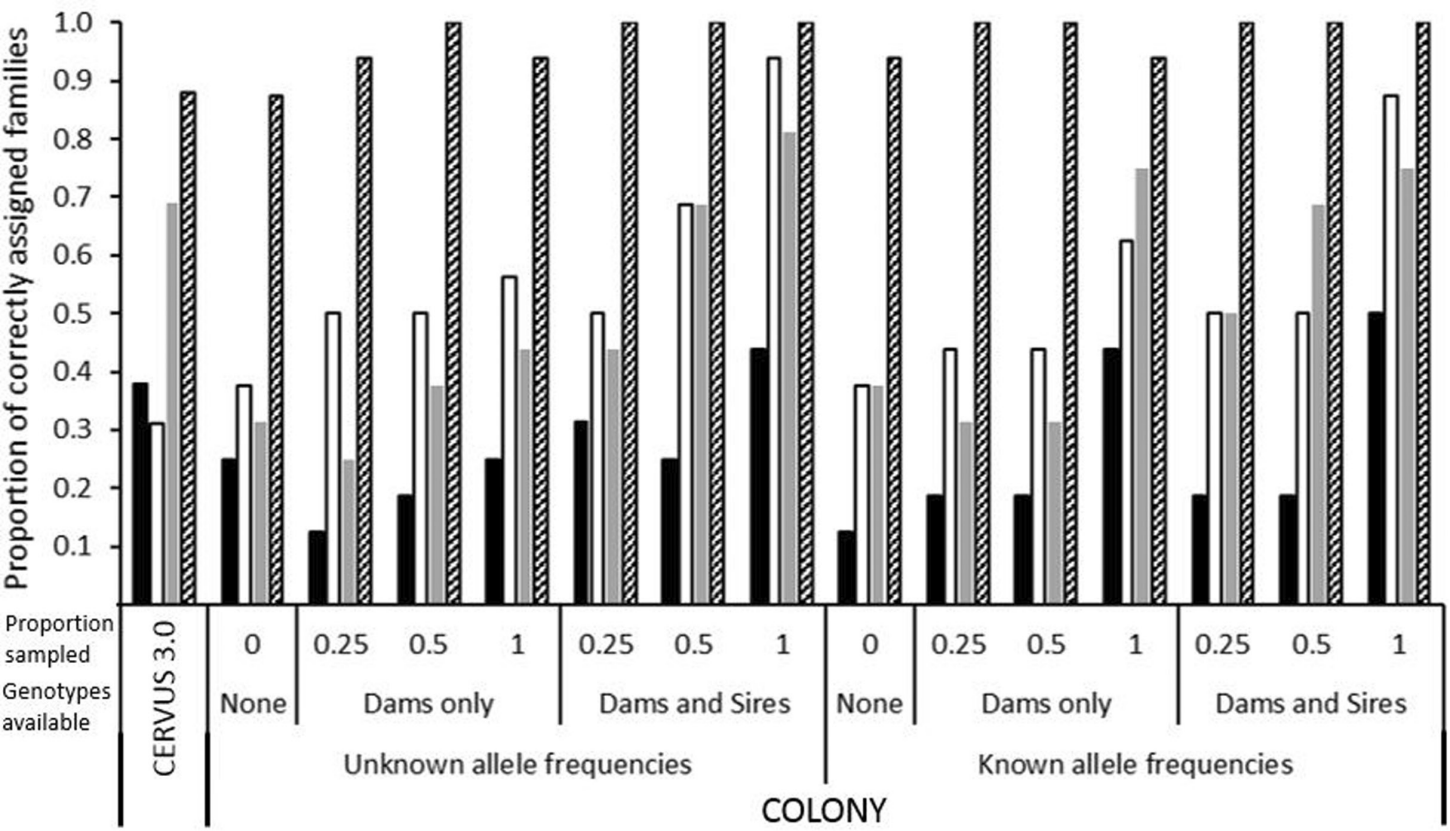
Proportion of the 16 family groups assigned correctly using CERVUS 3.0, when neither parent was known, and COLONY comparing the inference when allele frequencies were either known or unknown, depending on the type of genotype data available (none, dams only, or both dams and sires) as well as the proportion of the candidate parent population genotyped. Four microsatellite panels were compared: 5 loci with low polymorphism (5L; solid black), 5 loci with high polymorphism (5H; white), 7 loci (gray) and 11 loci (diagonal lines).

Both GERUD 2.0 and SLMM require family groups to be known *a priori* so were not applicable. However, since data can be presented to CERVUS 3.0 and COLONY without specifying family groups, it was of interest to observe the proportion of offspring that could be correctly assigned to each family (Figure 3).

The 11 microsatellite loci panel was able to correctly assign offspring to the family groups in 88%–100% of the scenarios run. In nine of these scenarios, COLONY was able to correctly assign 100% of offspring to their family groups when a pool of both sire and dam candidates were available irrespective of proportion sampled or known allele frequencies. On a few occasions, COLONY was also able to correctly assign family groups when only dams were known. The lowest proportions (88%) of correct assignment in both CERVUS 3.0 and COLONY occurred when no parental genotypes were specified. In all other scenarios, the family assignment rate was 94% (Figure 3).

Using all other reduced microsatellite panels, the correct family assignment rate significantly decreased to an average of 51%, 54%, and 27% for the 7, 5H, and 5L loci panels, respectively, using both CERVUS 3.0 and COLONY. In most cases, the 5H panel with the inclusion of the two most variable loci, was able to achieve better assignment rates than the 7 loci panel which had these markers removed.

Changing the stipulated proportion of the population that had been genotyped in COLONY did not affect the outcomes for the 11‐microsatellite panel, but it did have consequences on the outcomes from the reduced microsatellite panels. The largest differences were noted in the 5H loci panel when both dam and sire genotypes are available without known allele frequencies. A 50% correct family assignment rate was observed when a quarter of the parental population was said to be genotyped compared to a 94% correct family assignment when 100% of the parental population was genotyped. A similar rate of changed assignment rate was observed when only dam genotypes and known allele frequencies were specified for the 7 loci panel. In this case, when 100% of the dam population was said to be genotyped, 75% of offspring were correctly assigned to their family group. However, this was reduced to only 31% correct family assignment when it was specified only a quarter of the dam population was genotyped (Figure 3).

### 3.4 Proportion of offspring assigned to their correct parents

Following from the previous section and recalling these data were derived from 16 pair matings, it was also of interest to calculate the proportion of offspring correctly assigned not only to their family groups but also to their correct parents.

**FIGURE 4:**
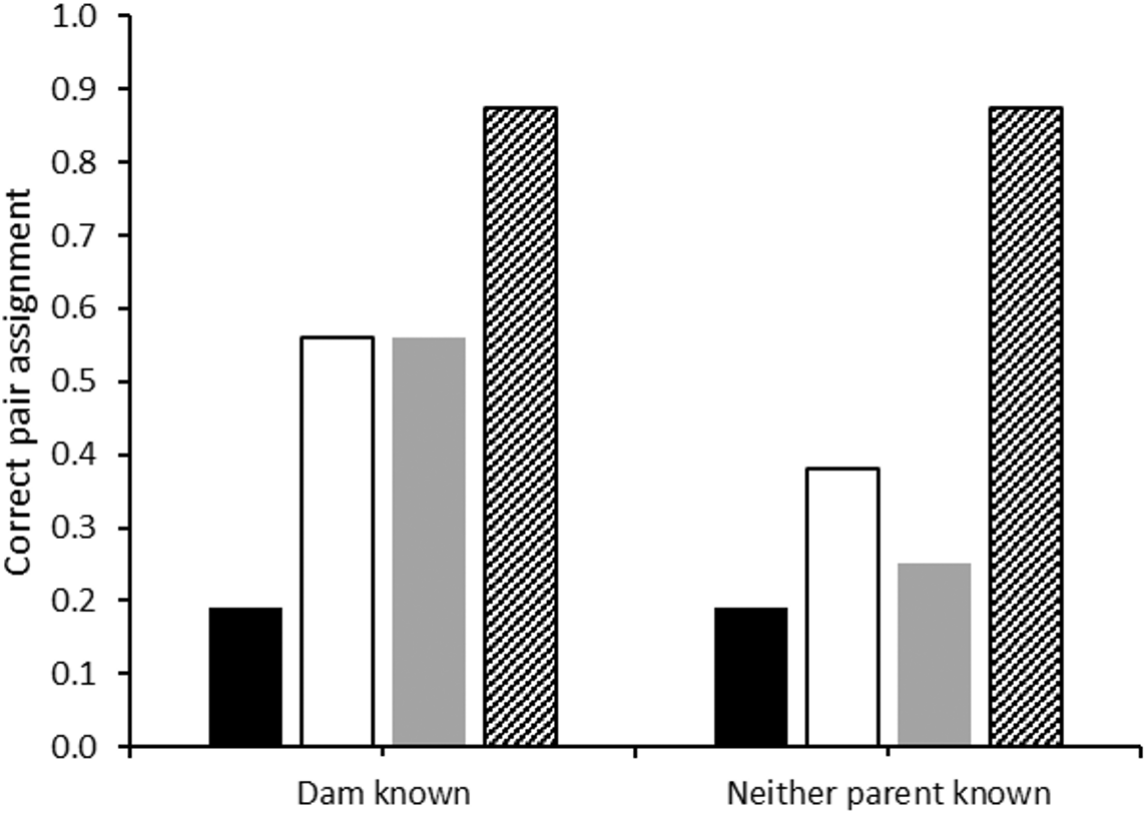
Proportion of the 16 family groups assigned to their correct parents using CERVUS 3.0 when both the dam was known and when neither parent was known. Four microsatellite panels were compared: 5 loci with low polymorphism (5L; solid black), 5 loci with high polymorphism (5H; white), 7 loci (gray) and 11 loci (diagonal lines).

As reported by Isberg et al. (2004), using the 11 loci panel in CERVUS 3.0, there were two individuals from two families that were incorrectly assigned to their parents when both the dams were specified as known and unknown, leading to a correct parent assignment rate in 14 out of 16 family groups (88%; Figure 4). Reducing the number of microsatellites in the panel had a definite effect on the correct parent assignment rate but was less significant when the dam genotype was known.

**FIGURE 5:**
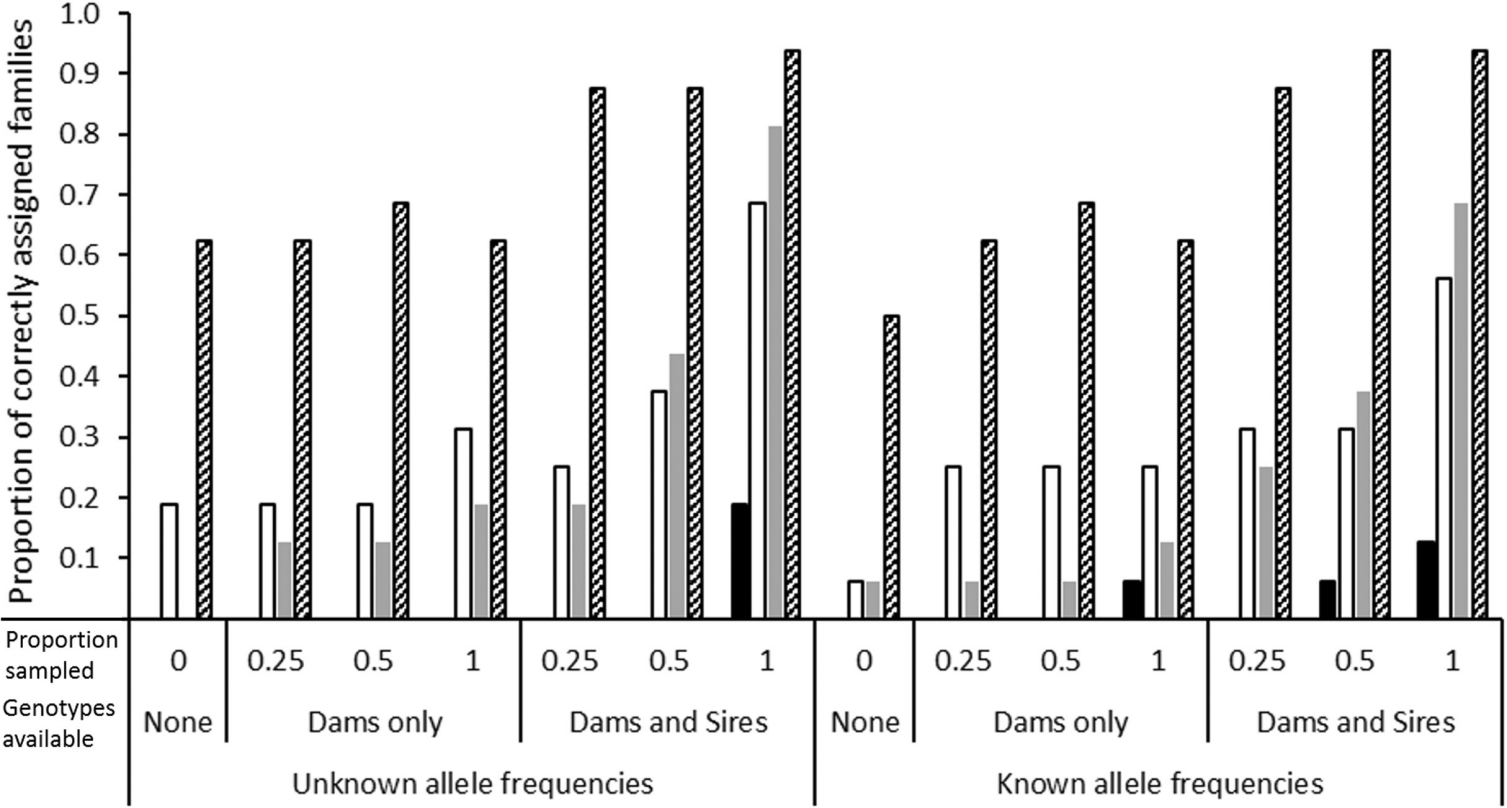
Proportion of the 16 family groups assigned correctly to their correct parents using COLONY comparing the inference when allele frequencies were either known or unknown, depending on the type of genotype data available (none, dams only, or both dams and sires) as well as the proportion of the candidate parent population genotyped. Four microsatellite panels were compared: 5 loci with low polymorphism (5L; solid black), 5 loci with high polymorphism (5H; white), 7 loci (gray), and 11 loci (diagonal lines).

Using the 5L loci panel, CERVUS 3.0 (Figure 4) could only assign 19% of the correct family groups to a specific pair regardless of whether the dam was known or unknown. Similarly, COLONY (Figure 5) was only able to correctly assign family to correct parentage in four of the 14 scenarios and, at best, at the same rate of assignment as CERVUS 3.0 (i.e., 19% when 100% of the parental genotypes were available). When no parental genotypes were known, COLONY could not assign any family group to one male and one female with the 5L panel.

The impact of specifying higher proportions of the population genotypes was particularly evident leading to an increase in correct parent assignment in most scenarios in COLONY (Figure 5). In general, specifying the allele frequencies led to slightly reduced rates of correct assignment.

### 3.5 Sibship assignment plots

**FIGURE 6:**
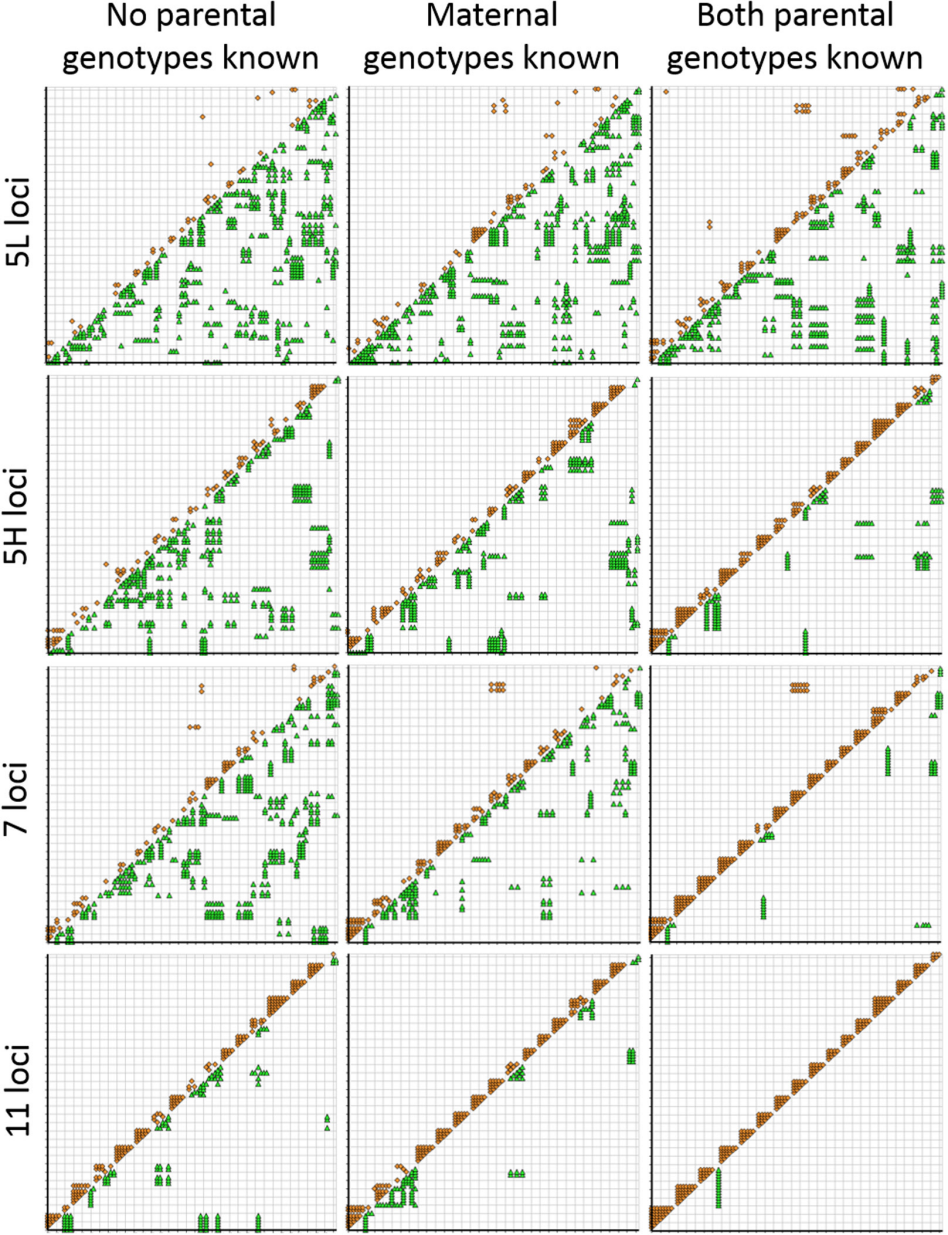
Sibship assignment plots based on the best maximum likelihood full‐pedigree analysis using COLONY (Wang, 2016) and 101 offspring genotypes derived from 16 known‐breeding pair family groups (Isberg et al., 2004). Different numbers of microsatellite loci were evaluated as well as specifying either no known parental genotypes, only maternal genotypes are known or both parental genotypes are known. *X*‐ and *Y*‐axes for each plot are offspring IDs in the same order. Full‐sibling relationships between two individuals are shown by an orange square above the diagonal, while half‐sib relationships are shown by a green triangle below the diagonal. Given these data are 16 family groups of full‐siblings, no half‐sibs should be present.

COLONY produces a graphical representation of each sibship assignment. A subset of the scenarios run in this study are shown in Figure 6 using the different microsatellite panels specifying no known parental genotypes, only maternal genotypes are known or both parental genotypes are known. Full sibs are represented by orange squares above the diagonal and half‐sibs by green triangles below the diagonal. As the number of microsatellite loci increases, combined with more parental genotype information, the greater the rate of full‐sib assignment. Among the Isberg et al. (2004) dataset of 16 full‐sib families, the closest sibship assignment was for 11 microsatellites with 100% of known parental genotypes (bottom right corner of Figure 6). Interestingly, in this scenario, one individual (indicated by the green below the diagonal) was assigned to the correct dam but to a sire who was housed on the opposite side of a driveway 20+ m away. Going back to the paternal genotypes and removing the dam alleles, COLONY had assigned the wrong sire (B2M) because he was homozygous at two more loci than the correct sire (B3M) and the maximum likelihood algorithm preferences homozygotes.

## 4 DISCUSSION

The different scenarios used herein clearly demonstrate how the underlying design of a study on crocodilian mating systems can influence the number of inferred sires and proportion of correct family assignments. In particular, when using software that does not require family groups to be designated *a priori*, more microsatellites were required along with more candidate parental genotypes to reduce the number of inferred sires toward the correct number, in this case one, and to achieve higher rates of correct family assignment.

These results are in turn reflective of the number of alleles within each microsatellite panel. In the case of the 7 loci panel used herein, it was decided to remove the two most and the two least polymorphic loci from the full panel leaving 35 alleles. By comparison, the 5H panel, which was constructed from the five most highly polymorphic loci, had 42 alleles. These two microsatellite panels produced more closely similar results than the other two panels (11 loci = 65 alleles; 5L loci = 23 alleles) but were not as accurate as the 11 loci panel. Counter to this, using GERUD 2.0 and the SLMM that designate family groups *a priori*, using less markers (5L panel) accurately inferred one true father regardless of whether the dam's genotype was known although less paternal genotypes were reconstructed when the maternal genotype was given. The reverse could also be possible whereby underestimation of multiple paternity may also occur with few, less informative markers.

From Table 1, when the total number of alleles for each crocodilian parentage analysis study are tallied, the number of alleles varies from 24 (Amavet et al., 2012; Hu & Wu, 2010) to 91 alleles (Oliveira et al., 2014) with an average of 42 total alleles equivalent to the 5H panel used herein. Following from the findings in this study and given that nest assignment is possible in crocodilians if offspring are being sampled from eggs/embryos, when fewer alleles are available, using GERUD 2.0 or the SLMM is sufficient to accurately infer sire number. However, neither of these methods are applicable if hatchlings are sampled in post‐hatching creches, such as those in Budd et al. (2015), where sibship/family assignments also need to be inferred. Further, the utility of SLMM and GERUD 2.0 is lowered when genotypic mismatches from mutations, null alleles, or experimental error are present so it is recommended that maternal genotypes be specified as well as knowledge of allelic frequencies within the adult population prior to parentage assignment using these programs. On the other hand, when a greater number of alleles are available more analysis options are available and the benefit of using more than one analysis package is evidenced herein (Figures 1, 2, 3, 4, 5, 6).

Despite these data being derived from single pair matings, up to seven sires could be inferred using COLONY and up to six using CERVUS 3.0. The commonality in these scenarios was when subsets of maternal and paternal genotypes were unknown and further emphasized when fewer alleles were presented. These scenarios also showed reduced proportions of correct family assignments (Figures 3, 4, 5, 6). Thus, the implications of the underlying study design need to be understood and even circumvented to avoid overinflated estimates of polygamy/polyandry being reported (Figures 1 and 2). Jones et al. (2010) state that “parentage analysis is normally applied to systems in which candidate parents can be collected, so most techniques assume that there will be a sample of adult genotypes”. However, of the 17 studies on crocodilian parentage literature available to date (Table 1), only four have a subset of both parental genotypes while seven studies do not have any parental genotypes to include in the analyses and are completely reliant on computation and microsatellite informativeness.

Only two crocodilian studies have so far been conducted on known pedigree structures (ie both dam and sire are known) to test the utility of microsatellites panels in assigning correct parentage (*Caiman latirostrsis*, Zucoloto et al., 2009; *Crocodylus porosus*, Isberg et al., 2004). Despite the proven utility of these entire microsatellite panels to differentiate parentage, authors of subsequent studies in these species have chosen to use reduced microsatellite panels (18%–69% fewer total alleles; Amavet et al., 2008, 2012; Lewis et al., 2013) without simulation of the reduced accuracy of parentage assignment particularly in relation to an unknown pedigree. In general, based on the results from this study and the parentage assignment software used, a reduction in microsatellite/allele number most likely also upwardly biases the estimated number of sires.

An easy way to reduce some of the algorithmic burden is to try to obtain maternal DNA. This can be achieved, as originally described in Davis et al. (2001), during displays of nest protection which is well‐characterized in crocodilians (e.g., Thorbjarnarson, 1996; Webb & Cooper‐Preston, 1989). While nest protecting behavior often assumes that the female is also the biological dam, and indeed was found to be the case in Davis et al. (2001) and Lance et al. (2009), mis‐assignments have also been reported. Using a captive population of *Caiman latirostris* with a documented Studbook pedigree (Verdade & Andrade, 2003), Zucoloto et al. (2009) found that two out of six nests were protected by a non‐biological female. In both of these instances, the nest‐protecting female was heavier in body mass than the biological female (L.M. Verdade, pers. comm. 2019). Neither of these larger females laid their own clutch of eggs throughout the study year and so protection of her potential nesting site can be excluded. It cannot be ruled out that the behaviorally assigned female was protecting a nesting site she had previously laid eggs in (i.e., site fidelity; Lance et al., 2009; Zajdel et al., 2019) as that information is not available. Alternatively, the nest‐protective behavior observed may be misinterpreted as (1) protection of their normal, non‐reproductive niche basking area, (2) “joint care” or “alloprotection” behavior; (3) a (programmed) response to human presence, or (4) a social (mal)adaptation of the captive environment (Zucoloto et al., 2009). Milián‐García et al. (2016) also described potential alloprotection in captive *Crocodylus rhombifer* but the protecting female was identified before oviposition and therefore this behavior may be independent of reproductive effort. Further studies have also identified either potential communal nesting of females (Amavet et al., 2012) or accidental mixing of clutches during collection (Ojeda et al., 2017). All of this information is crucial when constructing pedigrees, patterns of mating systems and paternity estimation, both in captivity and in the wild, so not collecting at least potential maternal genotypes can be limiting to the outcomes of the study.

Understandably, it is not always possible or safe to take a tissue or blood sample from any adult crocodilian. However, at the very least, maternal DNA was successfully extracted from the membrane of unfertilized eggs in the Chinese alligator (*Alligator sinensis*; Hu & Wu, 2010), fetal membranes of the Nile crocodile (*Crocodylus niloticus*; Nöthling et al., 2020), and from the eggshell matrix in birds (e.g., Egloff et al., 2009). Extraction of environmental DNA (eDNA) from soil samples in direct contact with the eggs when the nest is opened may also be a useful source of maternal DNA (Adams et al., 2019) from mucous residue which is present on eggs at the time of oviposition. Either of these methods has the potential to add genotypic informativeness into investigations of family groups, mating systems, and paternity determination.

Due to the retrospective nature of this study, the number of offspring could not be varied and could also have had implications on the ability of the parentage analysis to infer correct parentage if these were not from single sire matings. For parentage analysis using parental reconstruction, such as GERUD 2.0, Jones et al. (2010) recommended that more than 8–10 offspring per family are required for successful reconstruction. On this basis, Zajdel et al. (2019) excluded 120 *A. mississippiensis* nests because only one to eight eggs from each was collected and could have produced overly inflated sire numbers. Of the other crocodilian studies summarized in Table 1, all incorporated offspring numbers greater than the recommended 8–10 offspring unless unavoidable, for example, only three hatchlings survived in some of the nests analyzed by Oliveira et al. (2014) and Rossi Lafferriere et al. (2016). Most of these studies had also genotyped all resultant hatchlings from a nest and reported contributions of each inferred sire to the clutch. Skewed paternal contributions were reported in *A. mississippiensis* (Lance et al., 2009; Zajdel et al., 2019), *Caiman crocodilus* (Oliveira et al., 2014), *Crocodylus intermedius* (Rossi Lafferriere et al., 2016), *Crocodylus moreletti* (McVay et al., 2008), and *C. rhombifer* (Milián‐García et al., 2016; Table 1) but the only study so far that has looked at paternal contribution in both embryos and resultant hatchling is Zajdel et al. (2019). While arguably it is only the live hatchlings that have the potential to contribute their genes to the next generation, not including unhatched embryos could also bias sire contribution estimates and potentially also exclude less reproductively fit males who contribute to fertilization but whose embryos do not survive to hatch. With the exception of Budd et al. (2015), all of these studies have collected and incubated eggs in artificial environments of constant temperature and humidity. Thus, these embryos are not under natural selection pressures where in the wild it is well documented that flooding and predation are often the most likely cause of embryo mortality (for example, *A. mississippiensis*: Joanen & McNease, 1989; Kushlan & Jacobsen, 1990; *C. crocodilus*: Allsteadt, 1994; *Caiman crocodilus yacare*: Campos, 1993; *C. porosus*: Webb & Cooper‐Preston, 1989; *Caiman yacare*: Cintra, 1988; *Melanosuchus niger*: Villamarín‐Jurado & Suárez, 2007) neither of which can be determined by paternal genes. More recently, environmental contaminants could also be a non‐genetic threat to embryo mortality (Rotstein et al., 2002). Future studies wanting to quantify the contribution of various sires should consider genotyping all fertile embryos irrespective of hatching outcome to get a full understanding of the mating system involved as well as bolster offspring numbers available for parentage analysis. However, a cautionary note again is that unless the microsatellite panel was appropriately constructed and tested on a known pedigree, the estimates of contributing sires might also be overinflated.

Despite inferences of up to 10 sires (Lewis et al., 2013) and a large proportion of clutches reported with multiple sires, up to 100% (Milián‐García et al., 2016; Oliveira et al., 2010), there appears to be no advantage of polygamy/promiscuity in crocodilians. So far, there is no evidence of increased fertility (Zajdel et al., 2019), hatching success (Lewis et al., 2013), or hatchling size (Zajdel et al., 2019) in multiply sired nests. Indeed, the evidence is counter to this. In fact, given the high rates of embryo and hatchling mortality in the wild, the male strategy of multiple mating might be to “spread the risk” over multiple females to ensure genetic endurance. From the female's standpoint though, nest site fidelity was reported in *A. mississippiensis* (Elsey et al., 2008; Lance et al., 2009; Zajdel et al., 2019), *Crocodylus acutus* (Rossi Lafferriere et al., 2016), and *C. crocodilus* (Oliveira et al., 2014) along with a higher degree of mate fidelity (Budd et al., 2015; Rossi Lafferriere et al., 2016) and reduced incidence of multiple paternity (Lance et al., 2009). Nest site fidelity might represent the dam's “ideal” nesting location and the risk of losing this nesting site to another female is more important than seeking multiple partners for their offspring. A better understanding of what constitutes an “ideal” nest site for these females is required (e.g., nesting material and availability, surrounding water depth and/nursery habitat, various water parameters such as flooding, tidal, salinity, or ease of access and type of predators; Somaweera et al., 2013), along with embryo and post‐hatchling survival data.

Constructing an accurate understanding of a mating system is challenging even when the outcome is known as it was in this dataset. To aid in correct parentage assignment, due consideration to experimental design is necessary with the inclusion of parental genotypes, adequate number of polymorphic microsatellites as well as using more than one parentage analysis technique to compare outcomes. Prior evaluation of the microsatellite panel to be used should include knowledge of the polymorphic loci in the species/population being evaluated, with the aim to maximize the number of alleles included, as well as prior testing of their parentage determination accuracy using a known pedigree (eg pairs from zoo, farms, or other captive sources). It should also be remembered that the number of sires inferred in each clutch displaying multiple paternity are only “hypothetical” until the true sire(s) are identified and confirmed by genotype. Furthermore, from a conservation perspective, the finding of multiple paternity has relatively little context without being able to relate this back to offspring recruitment into the adult (breeding) population. While this study has focussed on crocodilians, the outcomes would also be applicable to multiple paternity studies of other wildlife species.

## AUTHOR CONTRIBUTIONS

**Sally Isberg:** Conceptualization (lead); data curation (lead); formal analysis (lead); investigation (lead); methodology (lead); project administration (lead); resources (lead); software (lead); validation (lead); visualization (lead); writing – original draft (lead); writing – review and editing (lead).

## CONFLICT OF INTEREST

The author declares no conflict of interest.

## FUNDING INFORMATION

This research did not receive any funding from either public, commercial, or not‐for‐profit agencies.

## Data Availability

The raw data used in this study can be accessed at https://doi.org/10.5061/dryad.0cfxpnw5b.
